# Microbiota and gastric cancer: from molecular mechanisms to therapeutic strategies

**DOI:** 10.3389/fcimb.2025.1563061

**Published:** 2025-06-03

**Authors:** Zhou Chen, Dacheng Jin, Jinjing Hu, Defeng Guan, Qizhou Bai, Yunjiu Gou

**Affiliations:** ^1^ Department of Thoracic Surgery, Gansu Provincial Hospital, Lanzhou, Gansu, China; ^2^ The Third Clinical Medical College, Lanzhou University, Lanzhou, Gansu, China; ^3^ National Health Commission (NHC) Key Laboratory of Diagnosisand Therapy of Gastrointestinal Tumor, Lanzhou, Gansu, China; ^4^ The First Clinical Medical College, Lanzhou University, Lanzhou, Gansu, China; ^5^ Reproductive Medicine Center, The First Hospital of Lanzhou University, Lanzhou, Gansu, China

**Keywords:** gastric cancer, microbiota, molecular mechanisms, tumor microenvironment, targeted therapy

## Abstract

Gastric cancer, a prevalent malignancy globally, is influenced by various factors. The imbalance in the gut microbiome and the existence of particular intratumoural microbiota could have a strong connection with the onset and progression of gastric cancer. High-throughput sequencing technology and bioinformatics analysis have revealed a close correlation between abnormal abundance of specific microbial communities and the risk of gastric cancer. These microbial communities contribute to gastric cancer progression through mechanisms including increasing cellular genomic damage, inhibiting DNA repair, activating abnormal signaling pathways, exacerbating tumor hypoxia, and shaping a tumor immune-suppressive microenvironment. This significantly impacts the efficacy of gastric cancer treatments, including chemotherapy and immunotherapy. Probiotic, prebiotic, antibiotic, carrier-based, dietary interventions, fecal microbiota transplantation, and traditional Chinese medicine show potential applications in gastric cancer treatment. However, the molecular mechanisms regarding dysbiosis of microbiota, including gut microbiota, and intra-tumoral microbiota during the progression of gastric cancer, as well as the therapeutic efficacy of microbiota-related applications, still require extensive exploration through experiments.

## Graphical Abstract

This article presented the role of microbiota in gastric cancer from multiple aspects, including from molecular mechanisms to therapeutic interventions, in order to provide some suggestions for future research on tumor mechanism and clinical treatment.

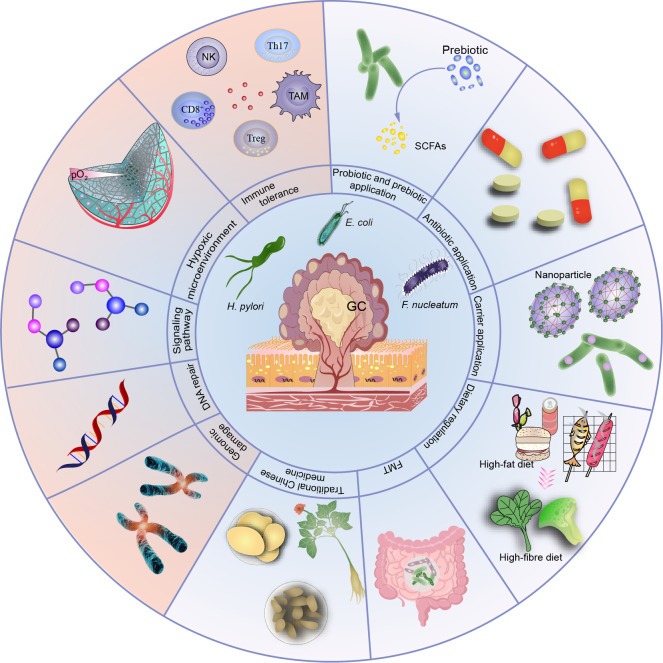

## 1 Introduction

The association between microorganisms and tumor genesis can be traced back to the 13th century (Pack, 1967). In the late 19th century, William Coley pioneered the use of a vaccine named “Coley’s toxins,” composed of two killed bacteria, *Streptococcus pyogenes* and *Serratia marcescens*, to treat various malignant tumors, resulting in promising therapeutic outcomes (Starnes, 1992). In the mid to late 20th century, some oncogenic viruses such as human herpesvirus 4 (HHV-4) and hepatitis B virus (HBV) were discovered (Epstein et al., 1964; Dane et al., 1970). Subsequently, the connection between microbiomics and tumor occurrence and development has aroused widespread interest among scholars. In particular, the advent of the first-generation sequencing method, the chain-termination approach, opened the door to deciphering the genetic code of life (Sanger et al., 1977). With advancements in sequencing technologies, such as next-generation sequencing, also known as high-throughput sequencing, and single-molecule, long-read sequencing, direct sequencing is possible. This has addressed issues of information loss and base mispairing, allowing us to better understand the structural composition of microbial communities (Radelof et al., 1998; Eid et al., 2009; Metzker, 2010). The microbial community, as an emerging field of research, has been found to exist in various types of tumors, including breast cancer, lung cancer, ovarian cancer, pancreatic cancer, and melanoma (Nejman et al., 2020). However, research on the microbial community in gastric cancer (GC) has only received widespread attention in the past decade. Dysbiosis of the microbial community may participate in the occurrence and development of GC through pathways such as activating inflammatory responses, influencing host immune systems, and interfering with cell signaling. Moreover, the structure of the microbial community is closely related to the efficacy of treatments such as chemotherapy and radiotherapy, as certain microbes may affect drug metabolism, absorption, and resistance. Therefore, some studies are attempting to target the microbial community for GC treatment, aiming to improve the tumor microenvironment (TME), enhance immune suppression, and increase drug efficacy by modulating the microbial community. Overall, research on the microbial community in GC is in a stage of vigorous development, requiring further in-depth studies and clinical validation, which are crucial for elucidating the pathogenesis of GC, identifying new therapeutic targets, and formulating personalized treatment strategies.

## 2 GC and alterations in the microbiota structure

GC is closely associated with alterations in the microbial community structure. Current research focuses on dysbiosis in the oral, gastric, and colonic microbiota (**Table 1**). These studies suggest that changes in the microbial community may play a significant role in the occurrence and development of GC, offering new insights into its prevention and treatment.

**Table 1:** Studies of microbiota related to GC.

Specimen	Sample size (tumor vs non-tumor)	Sequencing methods	Elevated microbiota	Decreased microbiota	Ref
Gastric mucosa	54, 81	16S rRNA gene sequencing	Actinobacteria, Bacillota	*Bacteroidota*, *Fusobacteria* spp.	(Federici et al., 2022)
Gastric juice	50, 45	16S rRNA gene sequencing	Neisseria sicca, Prevotella melaninogenica, Veillonella parvula, Prevotella jejuni, Veillonella atypica	–	(Wang et al., 2022)
Gastric cancer and adjacent noncancerous tissues	45, 45	Internal transcribed spacer rDNA gene analysis	Candida, Alternaria Fusicolla acetilerea,	*Saitozyma*, *Thermomyces*	(Zhong et al., 2021)
Gastric cancer and gastric mucosal tissue	53, 30	16S rRNA gene sequencing	*Prevotella* spp., *Oceanobacter, Methylobacterium*, *Syntrophomonas*, *Acinetobacter*, *Deiftia*	*Helicobacter*, *norank furibaculaceae*, *Escherichia-Shigella*, *Bifdobacterium*	(Peng et al., 2022)
Saliva	99, 194	16S rRNA gene sequencing	*Corynebacterium*, *Streptococcus* spp.	*Bulleidia*,*Fusobacterium*, *Peptostreptococcus*, *Lachnoanaerobaculum*,*Haemophilus*, *Neisseria* spp., *Parvimonas*, *Porphyromonas*, *Prevotella* spp.	(Huang et al., 2021)
Tongue coating	57, 80	16S rRNA gene sequencing	Bacillota	Bacteroidota	(Wu et al., 2018)
Mouthwash	165, 323	Shotgun metagenomic sequencing	*Betaproteobacteria*, *Neisseriales*, *Neisseriaceae*, *Neisseria mucocosa*, *Prevotella pleuritidis*	*Mycoplasma orale*, *Tenericutes*, *Eubacterium yurii*, *Cutibacterium*	(Yang et al., 2022b)
Saliva	10, 118	16S rRNA gene sequencing	Neisseria spp., Porphyromonas gingivalis	–	(Kageyama et al., 2019)
Tongue coating	11, 27	16S rDNA sequencing	Neisseria spp., Prevotella spp., Veillonella, Porphyromonas, Leptotrichia	Sphingomonas, Rudaea, Phyllobacterium, Helicobacter, Bradyrhizobium, Arthrobacter	(Wu et al., 2021)
Saliva and tongue coating	26, 18	2b-RAD sequencing	Malassezia globosa	Saccharomyces cerevisiae	(He et al., 2023)
Fecal samples	49, 49	16S rRNA gene sequencing	*Lactobacillus*, *Fusobacterium*, *Streptococcus* spp.	*Lachnospiraceae*, *Clostridia*, *Roseburia*, *Blautia*, *Actinobacteria*	(Yu et al., 2021)
Fecal samples	50, 56	Shotgun metagenomics sequencing	*Streptococcus* spp., *Prevotella* spp., *Veillonella*, *Lactobacillus*	–	(Erawijantari et al., 2020)

### 2.1 Intratumoural microbiota

The gastric microbiota plays a crucial role in maintaining endocrine balance, immune modulation, and promoting digestion and absorption. Significant alterations in the microbial composition and abundance occur in GC tumor tissues, leading to a state of microbial dysbiosis. This imbalance is widely considered to be a result of decreased microbial diversity and increased pathogenic microorganisms, among which *Helicobacter pylori* (*H. pylori*) infection is closely related. *H. pylori* is widely recognized as one of the most dangerous infection factors associated with GC, with a global infection rate exceeding 50%, and 1% to 3% of *H. pylori*-infected individuals developing GC (Noto et al., 2019; Xiao and Ma, 2022). *H. pylori* infection can result in the enrichment of other bacterial phyla, such as *Proteobacteria*, *Bacillota*, and *Bacteroidota* (Gao et al., 2018). On one hand, *H. pylori* has been demonstrated to activate NF-κB and induce the production of β-defensin in gastric epithelial cells through the cytotoxin-associated gene A protein (CagA), potentially influencing the microbiota composition (Hamanaka et al., 2001; Wada et al., 2001; Brandt et al., 2005). On the other hand, *H. pylori* directly inhibits acid secretion in epithelial cells through T4SS, CagA, and NF-κB-dependent mechanisms, leading to an increase in gastric pH. This less acidic environment promotes microbial diversification in the ecological niche, potentially enhancing diversity and reshaping the community structure of the gastric microbiota (Göõz et al., 2000; Saha et al., 2008; Smolka and Backert, 2012). Additionally, studies by Noto et al. have found that changes in the gastric microbiota are dependent on CagA and are not related to inflammatory responses, suggesting that CagA itself directly influences the microbial community structure (Noto et al., 2019). Moreover, in Drosophila intestinal stem cells, the expression of CagA fosters excessive cell proliferation and triggers the expression of innate immune components, such as Diptericin and Duox, which have the potential to modify the host microbiota (Jones et al., 2017). *H. pylori* also expresses the duodenal ulcer-promoting gene A (DupA), closely linked to peptic ulcer disease but exerting minimal impact on the microbiota, thereby preserving the relative abundance of the gastric microbial community. However, DupA(-) *H. pylori* is abundant in precancerous lesions (Chen et al., 2023a). Other studies suggest that although microbial diversity decreases in GC tissue, this result appears unrelated to *H. pylori*. In GC tissue, the abundance of *H. pylori* decreases, while other bacterial genera, such as *Citrobacter*, *Achromobacter*, *Clostridium*, *Prevotella* spp., *Rhodococcus*, *Propionibacterium acnes* (*P. acnes*), *Clostridium*, *Slackia exigua*, *Fusobacterium*, *Parvimonas micra*, *Streptococcus* spp., and *Dialister pneumosintes*, increase in TME, with most of these genera representing intestinal symbionts (Coker et al., 2018; Ferreira et al., 2018; Hsieh et al., 2018; Liu et al., 2019; Dai et al., 2021; Png et al., 2022; Zhou et al., 2022). Moreover, in patients with favorable prognosis, the presence of *H. pylori* in TME is significantly increased (Yang et al., 2023b). Another study indicates that *H. pylori* infection may diminish the efficacy of immune checkpoint inhibitor therapy, resulting in markedly shorter median progression-free survival (PFS) and overall survival (OS) among *H. pylor*i-positive patients. Consequently, long-term dynamic monitoring becomes essential for individuals with *H. pylori* infection (Magahis et al., 2023). Certain bacterial genera such as *Bacteroidota* and *Fusobacteria* spp., *Prevotella* spp., show variable abundance in tumor tissue (Ferreira et al., 2018; Liu et al., 2019). *Fusobacteria* spp. and *Prevotella* spp. are significantly associated with poorer overall survival in GC patients (Lehr et al., 2023). Interestingly, *Lactobacillus* sp*ecies (spp.)*, a probiotic, are significantly enriched in GC tissue, especially in the absence of *H. pylori* (Ferreira et al., 2018; Hsieh et al., 2018; Gantuya et al., 2020; Dai et al., 2021). This may be related to the elevated expression of IL-1β, mucin 4, and mucin 13 in gastric mucosa (Ferreira et al., 2018; Breugelmans et al., 2022; Kim et al., 2022). However, the exact role of probiotics in the TME of GC tissue warrants further investigation.

In GC patients with concomitant bile reflux, the gastric microbiota is significantly altered. These patients exhibit an enrichment of bacterial genera such as *Comamonas*, Pseudomonas, *Halomonas*, *Arthrobacter*, *Bradymonas*, *Shewanella*, and *Marinobacter* (Huang et al., 2022). This phenomenon is likely attributed to the presence of bile acids (BAs), which include free and conjugated forms. The presence of BAs in gastric fluid, such as deoxycholic acid (DCA), reduces microbial diversity and leads to significant enrichment of *Limosilactobacillus*, *Burkholderia-Caballeronia-Paraburkholderia*, and *Rikenellaceae RC9* (Xu et al., 2023b). Conjugated BAs elevate gastric pH, promoting the proliferation of bacteria producing lipopolysaccharide (LPS) in the stomach. As a result, the relative abundance of bacteria such as *Neisseria sicca*, *Veillonella parvula*, *Veillonella atypica*, *Prevotella melaninogenica*, and *Parvimonas pallens* significantly increases in gastric fluid (Wang et al., 2022). This has a profound impact on patients who have undergone gastrointestinal reconstruction surgery.

Some bacterial genera previously unreported in the gastric microbiota have been identified, such as *Keratinibaculum* spp., an anaerobic thermophilic bacterium isolated from soil (Mannion et al., 2023).

In GC tissues, fungal dysbiosis is observed, with *Candida albicans* serving as a biomarker for GC. The abundance of *Candida albicans* significantly increases in GC, reshaping the microbial composition. This is characterized by an elevated presence of filamentous fungi such as *Fusicolla acetilerea*, *Fusicolla aquaeductuum*, and *Arcopilus aureus*, while *Candida glabrata*, *Saitozyma podzolica*, *Penicillium arenicola*, and *Aspergillus montevidensis* exhibit markedly reduced abundance (Zhong et al., 2021). These alterations in microbial composition, predominantly featuring certain pathogenic bacteria, impact the prognosis of GC. For example, the heightened presence of *Methylobacterium* in GC tissues is significantly linked to an unfavorable prognosis in GC patients (Peng et al., 2022). A correlation analysis of the gastric mucosal microbiome in 170 GC tumor tissues and matched non-tumor tissues with immune-activated related transcripts revealed that *Akkermansia muciniphila* may play a role in modulating the expression of Granzyme B in the gastric cancer mucosal microenvironment. However, this requires further exploration (Lu et al., 2024). In addition, the GC microbiome was classified into three distinct subtypes (MS1, MS2, and MS3): MS1 exhibited high immune activity and enrichment of microbiota associated with immunotherapy and butyrate production, suggesting a potential sensitivity to immunotherapy; MS2 showed the highest α-diversity and activation of the TFF signaling pathway; MS3 was characterized by epithelial-mesenchymal transition (EMT), associated with poor prognosis and reduced responsiveness to chemotherapy. These findings provide novel insights into the relationship between GC microbiome characteristics, prognosis, and treatment efficacy, contributing to the development of personalized therapeutic strategies (Wang et al., 2024).

### 2.2 Oral microbiota

The functions of oral microbiota in oral health include maintaining the health of oral mucosa, participating in food digestion, regulating oral pH balance, and resisting invasion by external pathogenic microorganisms. When the oral microbial community becomes imbalanced, it not only leads to the occurrence of oral diseases but also correlates with the risk of GC. In fact, abundant oral bacteria, such as *Peptostreptococcus*, *Streptococcus* spp., *Fusobacterium*, and *Campylobacter concisus*, can be detected in GC samples and may serve as potential non-invasive biomarkers (Chen et al., 2019; Cui et al., 2019; Feng et al., 2023). Oral-associated microbial communities, including *Veillonella parvula* and *Streptococcus oralis*, are enriched in gastric cancer tissues and are associated with overall survival (Lei et al., 2024). The changes in microbial composition are characterized by the accumulation of pro-inflammatory bacteria such as *Corynebacterium* and *Streptococcus* spp., and a reduction in bacteria metabolizing carcinogenic substances like *Haemophilus* and *Neisseria* spp (Wu et al., 2018; Huang et al., 2021). However, in other studies, *Neisseria* spp. and *Prevotella* spp. are significantly enriched, while *Mycoplasma* and *Eubacterium* are reduced (Kageyama et al., 2019; Yang et al., 2022b). The ectopic colonization of oral microbiota may drive dysbiosis in the microbial ecology of GC tissue infected with *H. pylori* (Wu et al., 2021). Furthermore, fungal dysbiosis has been observed in the oral microbiome. For instance, samples of saliva and tongue coating collected from GC patients are enriched with *Malassezia globosa*, while *Saccharomyces cerevisiae* is reduced (He et al., 2023). Tongue coating displays varying colors and thicknesses, each harboring distinct microbial communities. Bacteria such as *Capnocytophaga leadbetteri*, fungus *Ampelomyces_sp_IRAN_1* could potentially serve as biomarkers for the white thin coating, while *Megasphaera micronuciformis*, *Prevotella maculosa*, *Acinetobacter ursingii*, and *Selenomonas* sp*utigena ATCC 35185* may serve as biomarkers for the white thick coating (Xu et al., 2019). This provides a novel approach to tongue coating diagnosis.

### 2.3 Fecal microbiota

The occurrence of GC is intricately linked to the composition and dynamics of the gut microbiota. Under normal circumstances, the gut microbiota plays a critical role in maintaining intestinal homeostasis and overall host health. It contributes to various physiological processes, including nutrient metabolism, immune system regulation, and protection against pathogenic invaders. However, when the gut microbiota is imbalanced, it may trigger chronic inflammation, affect the host’s immune system, and thereby increase the risk of GC. For example, fecal *Streptococcus* spp. alterations are closely linked to GC incidence and liver metastasis, suggesting their potential as biomarkers for GC prediction. These findings offer valuable insights into early diagnosis and treatment strategies for GC (Yu et al., 2021; Chen et al., 2022a). In animal models, the abundance of the phyla *Actinobacteria* and *Bacillota* is highest in the GC group (Yu et al., 2020). The intestinal microbiota composition of invasive GC patients infected with *H. pylori* has changed, characterized by a significant reduction in protective bacterial genera such as *Lactobacillus* (Devi et al., 2021). Additionally, post GC surgery patients exhibit higher species diversity and richness in their intestinal microbiota, along with increased abundance of aerobic, facultative anaerobic bacteria, and oral microbiota, indicating an association with postoperative complications such as the occurrence of metachronous colorectal cancer after gastric resection (Erawijantari et al., 2020). Certain intestinal microbial communities can differentiate between surgical and non-surgical GC patients, including *Enterococcus*, *Corynebacterium*, *Megasphaera*, *Roseburia*, and *Lachnospira.* GC patients with lymph node metastasis show no significant differences compared to those receiving chemotherapy. Furthermore, the abundance of *Blautia*, *Oscillospira*, and *Ruminococcus* is associated with Ki67 expression, while the abundance of *Prevotella* spp., *Lachnospira*, *Eubacterium*, and *Desulfovibrio* correlates with HER2 expression (Chen et al., 2022a). The dysbiosis of microbial communities in GC patients involves the enrichment or reduction of multiple microbial taxa, and the identification of representative microbes remains challenging. In an *in vivo* GC model, the colonization of *Enterotoxin Bacteroides fragilis* in the mouse intestines significantly accelerated chemotherapy-induced muscle and adipose tissue depletion, and promoted the development of GC cachexia by disrupting cell junctions and attracting M1 macrophages, thereby damaging the intestinal mucosal barrier (Wu et al., 2024a).

## 3 The pro-carcinogenic mechanisms of microbiota in GC

The dysregulation of the microbial community contributes to the complex mechanisms underlying the initiation and progression of GC. Current research indicate that dysbiotic microbiota can drive tumorigenesis and progression by enhancing host genomic damage, impeding cellular DNA repair, activating aberrant cellular signaling pathways, influencing tumor cell metabolism, and reshaping the tumor immune microenvironment.

### 3.1 Microbiota dysbiosis and gastric epithelial cell genomic damage

**Figure 1:**
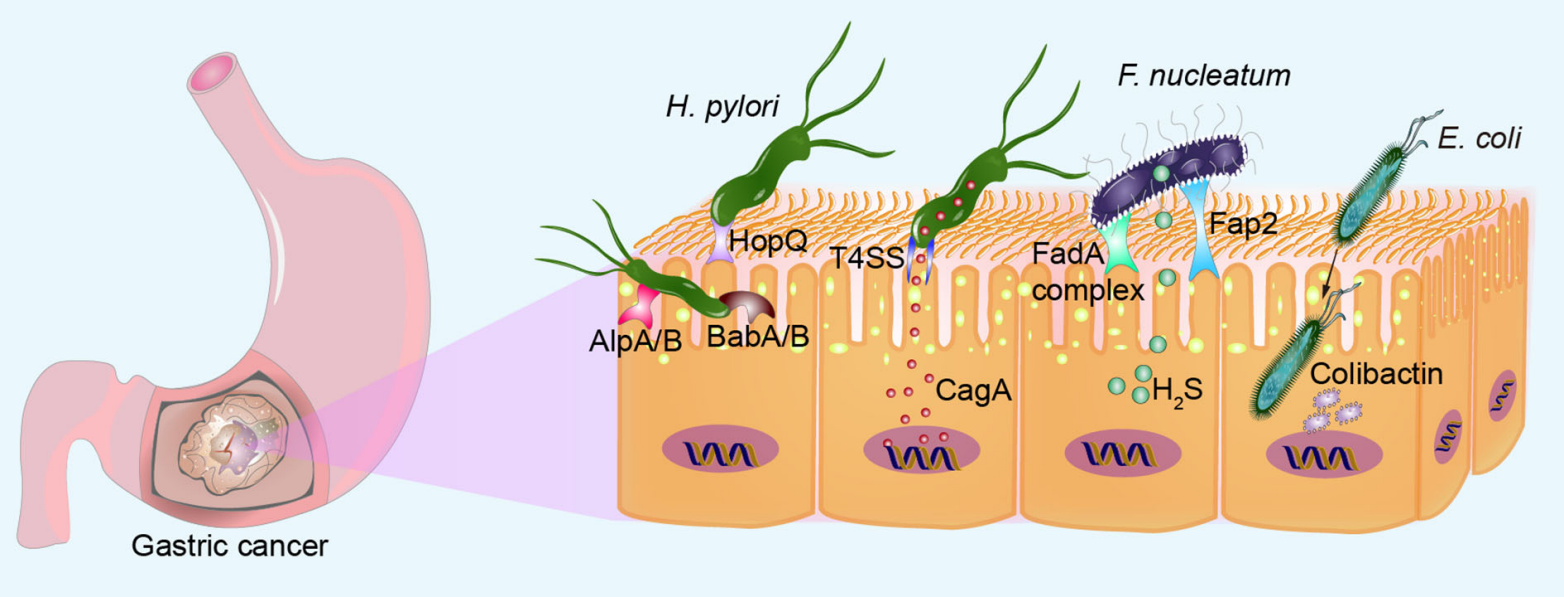
Microbial adhesion and invasion of gastric epithelial cells. *H. pylori* can bind to gastric epithelial cells via adhesin HopQ, glycan-modified proteins AlpA/B and BabA/B. *H. pylori* directly injects a potent virulence protein CagA into epithelial cells via the T4SS. *F. nucleatum* adheres to gastric epithelial cells through the pre-FadA-mFadA complex and Fap2 galactose-binding lectin, ensuring bacterial invasion of host cells. *F. nucleatum* produces high levels of H_2_S, increasing DNA damage. Once internalized by host cells, *E. coli* secretes genotoxin colibactin, inducing DNA double-strand breaks.

In its quest for long-term residence in the host stomach, *H. pylori* employs a diverse array of outer membrane adhesins to optimize its binding to the gastric mucosa. These adhesins facilitate a strong and persistent interaction with the host epithelial cells, promoting the bacterium’s survival and persistence in the gastric environment (**Figure 1**). *H. pylori* attaches to gastric epithelial cells using adhesins like HopQ and carcinoembryonic antigen-related cell adhesion molecules (Odenbreit et al., 2000; Javaheri et al., 2016; Hamway et al., 2020). Key adhesins in *H. pylori*, such as AlpA/B and BabA/B, are glycosylated, enhancing their binding ability. Loss of glycosylation severely impairs adhesin resistance to proteases, stability, and binding capacity (Teng et al., 2022). Upon binding to host epithelial cells, the cag pathogenicity island encodes a bacterial type IV secretion system (T4SS) that delivers a potent virulence protein, CagA, directly into epithelial cells. This event affects multiple pathways in host cells, stimulating epithelial cell proliferation and contributing to gastric carcinogenesis (Odenbreit et al., 2000; Ohnishi et al., 2008; Javaheri et al., 2016; Noto et al., 2019; Hamway et al., 2020). Mechanistically, CagA leads to aberrant β-catenin activation, promoting GC cell proliferation (Franco et al., 2005). Treatment of *H. pylori*-infected mice with the β-catenin inhibitor (KYA1797A) could significantly alleviate gastric epithelial DNA damage (Li et al., 2023). *Escherichia coli* (*E. coli*) and *Fusobacterium nucleatum* (*F. nucleatum*) possess a unique bacterial adhesin/invasin called FadA, which presents in two distinct states: pre-FadA and mature FadA (mFadA). Initially, pre-FadA is embedded within the inner membrane and remains soluble under neutral pH conditions. Upon maturation, mFadA becomes insoluble and is subsequently secreted outside the bacterium. When fluorescently labeled mFadA is introduced to epithelial cells alone, no binding is detected. However, when combined with unlabeled pre-FadA, binding and invasion of epithelial cells by mFadA occur. The Pre-FadA-mFadA complex could anchor within the inner membrane and extend through the outer membrane, facilitating bacterial invasion of host cells (Xu et al., 2007). Once internalized by host cells, *E. coli* secretes the genotoxin colibactin, leading to crosslinking between induced DNA strand and double-strand DNA breaks (Cullin et al., 2021). *F. nucleatum* utilizes lectin-like adhesins and a “zipping” mechanism to adhere to and invade human gingival epithelial cells (Han et al., 2000), or interacts with the Gal-GalNAc carbohydrate moiety on cell surfaces through its Fap2 galactose-binding lectin, specifically colonizing colorectal cancer and breast cancer (Abed et al., 2016; Parhi et al., 2020). This interaction may induce EMT, a critical process associated with cancer cell invasion, metastasis, stemness, and therapeutic resistance (Zhang et al., 2020). *F. nucleatum* can generate significant quantities of hydrogen sulfide (H_2_S) from L-cysteine via the enzymatic activity of L-cysteine desulfhydrase, leading to increased DNA damage (Fukamachi et al., 2002; Basudhar et al., 2016). Evidence suggests that the production of H_2_S contributes to DNA damage, partly through the generation of reactive oxygen species (ROS) (Attene-Ramos et al., 2010). The invasion of oral epithelial cells by *Prevotella intermedia* requires type C fimbriae, which are highly enriched in GC tissue (Dorn et al., 1998). Further research is needed to determine if *Prevotella intermedia* invades GC cells in the same manner and to elucidate the specific molecular mechanisms involved.

Pathogenic bacteria can also generate carcinogens through the metabolism of dietary components. Chronic *H. pylori* infection reduces gastric acid secretion, potentially fostering the growth of diverse gastric bacterial communities. The alteration in the microbiota could enhance aggression towards the gastric mucosa, potentially culminating in malignant tumor formation. The microbiota sustains inflammation and converts nitrate to N-nitroso compounds, thereby promoting malignancy. The functional composition of the overall GC microbiota demonstrates an augmented presence of enzymes such as nitrate reductase, which catalyzes the reduction of nitrate to nitrite, and nitrite reductase, facilitating the conversion of nitrite to nitric oxide (Ferreira et al., 2018). This increased enzymatic activity suggests a potential mechanism through which the microbiota contributes to the pathogenesis of GC. Elevated protein intake can result in heightened levels of protein within the colon. In this environment, various bacteria, including certain *Bacillota* and *Bacteroidota*, metabolize amino acids into N-nitrosyl compounds. These compounds can induce DNA alkylation and host mutations, potentially contributing to carcinogenesis (Gill and Rowland, 2002). Colonic bacteria metabolize carcinogens, generating compounds that damage DNA, such as ethanol and heterocyclic amines, or directly producing carcinogens like non-hexane (Huycke and Gaskins, 2004). Primary bile acids are converted into secondary deoxycholic acid (DCA) by certain bacteria, including *Clostridium scindens*. DCA disrupts cell membranes, releasing arachidonic acid as a tumor promoter. Arachidonic acid, when metabolized by COX-2 and lipoxygenase, undergoes conversion into prostaglandins and ROS. These compounds play a crucial role in triggering inflammatory responses and causing DNA damage, contributing to various pathological conditions. Furthermore, taurocholic acid serves as a tumor promoter by fostering the production of genotoxic hydrogen sulfide and fueling the expansion of specific inflammatory bacteria, such as *Bilophila wadsworthia*, contributing to carcinogenesis (Ridlon et al., 2016). Under conditions of iron deficiency, *H. pylori* exacerbates gastric injury in insulin-gastrin mice, highlighting the interplay between bacterial infection and nutrient status in gastrointestinal health. While the observed phenotypes are not mechanistically driven by changes in the gastric microbiota, targeted metabolomics studies unveiled substantial alterations in bile acids among iron-deficient mice infected with *H. pylori*. Notably, the carcinogenic bile acid DCA showed significant upregulation. Treatment with DCA worsened the severity of gastric injury in *H. pylori*-infected mice. *In vitro* experiments demonstrated that DCA enhances the translocation of the *H. pylori* oncogenic protein CagA into host cells (Noto et al., 2022). TDCA and LPS drive gastric carcinogenesis by triggering activation of the IL-6/JAK1/STAT3 pathway in gastric epithelial cells, implicating inflammation in the development of GC (Wang et al., 2022). Conversely, DCA induces alterations in the gastric environment, characterized by abnormal bile acid metabolism and microbial dysbiosis. Specifically, there is a notable enrichment of *Gemmobacter* and *Lactobacillus*, suggesting a complex interplay between bile acids and the gastric microbiota in gastric pathophysiology (Jin et al., 2022). Bile acids, shown to function as endogenous antagonists of leukemia inhibitory factor (LIF), bind to a heterodimeric receptor during tumor initiation. Tissue analysis of bile acid content in both non-cancerous and GC biopsies demonstrates an accumulation of bile acids within cancer tissues. Specifically, glycodeoxycholic acid acts as a negative regulator of LIFR expression (Di Giorgio et al., 2024).

### 3.2 Microbiota dysbiosis and gastric epithelial cell genome repair

**Figure 2:**
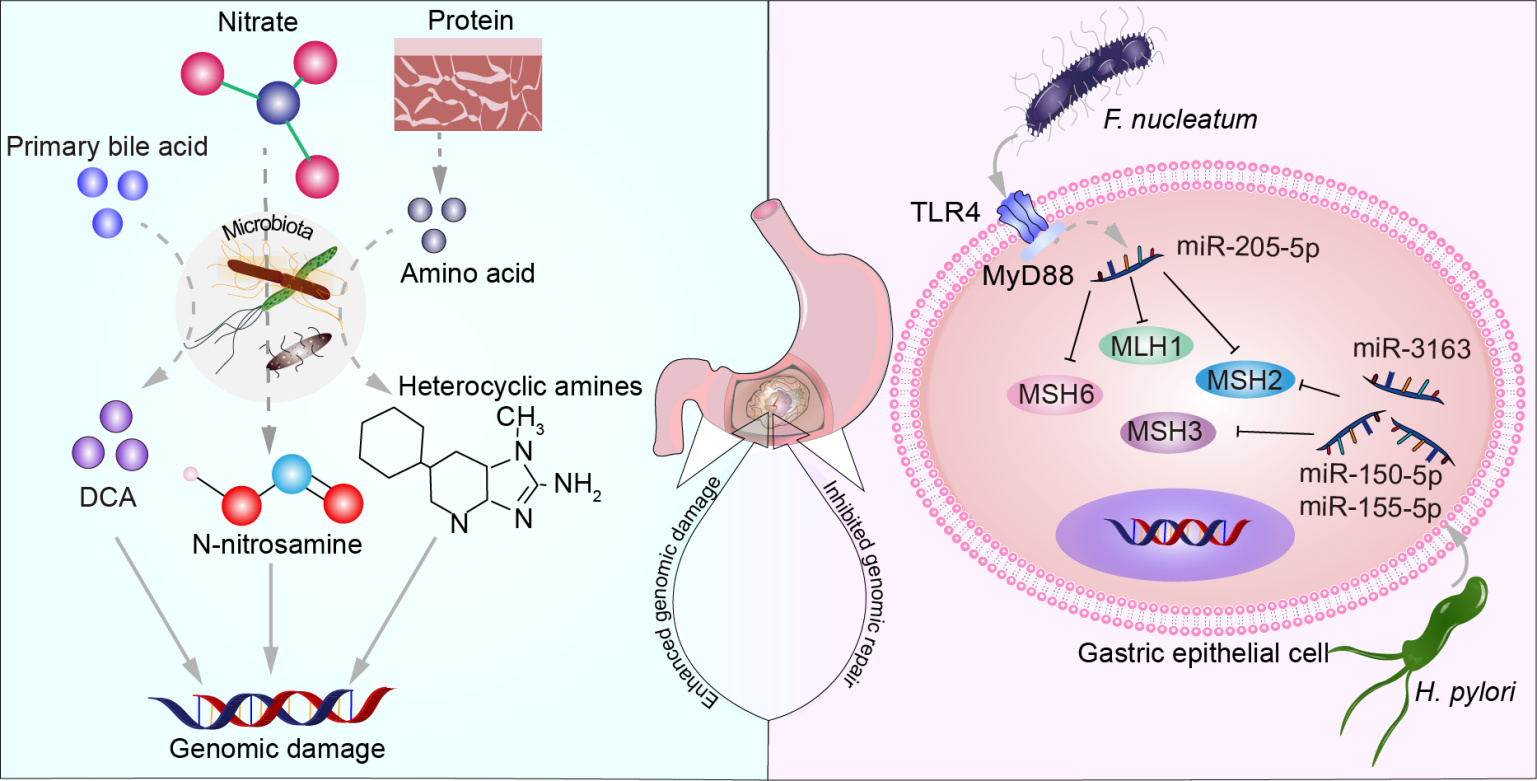
The microbiota increases host cell genomic damage and suppresses genome repair. Primary bile acids, nitrate, and proteins metabolized by certain microbial communities produce substances such as DCA, N-nitrosamines, and heterocyclic amines, leading to DNA damage. *F. nucleatum* increases the expression of miR-205-5p through the TLR4 and MyD88-dependent innate immune signaling pathway, suppressing the expression of MLH1, MSH2, and MSH6. *H. pylori* upregulates miR-150-5p, miR-155-5p, and miR-3163 to suppress the expression of MSH2 and MSH3 proteins.

Cells undergo a series of complex biological processes to repair DNA when subjected to external damage or internal errors resulting in DNA breaks, base damage, and other situations, thereby preserving the genome’s integrity and stability. These processes primarily include direct damage repair mechanisms such as mismatch repair (MMR), single-strand break repair (SSBR), and double-strand break repair (DSBR). Additionally, they encompass indirect damage repair mechanisms like nucleotide excision repair (NER), base excision repair (BER), cross-link repair. MMR is a highly conserved biological pathway crucial for maintaining genome stability. This pathway specifically targets base mispairs and insertion/deletion mispairs that arise during DNA replication and recombination processes (Li, 2008). Co-culturing GC cells with various strains of *H. pylori* results in a dose-dependent decrease in the levels of MMR proteins, including MutS (MSH2 and MSH6) and MutL (MLH1, PMS2, and PMS1) (Kim et al., 2002). This may be attributed to CagA EPIYA motifs and vacuolating cytotoxin A (vacA) genotypes (Mi et al., 2020). *H. pylori* suppresses the expression of MMR proteins by upregulating miR-155-5p, miR-3163, and miR-150-5p (Santos et al., 2017). *F. nucleatum* triggers the expression of miR-205-5p by activating the Toll-like receptor 4 (TLR4) and MyD88-dependent innate immune signaling pathway. This upregulation, in turn, suppresses the expression of key MMR proteins (MLH1, MSH2, and MSH6). The resulting MMR deficiency leads to increased DNA damage and enhanced cell proliferation, contributing to the progression of squamous cell carcinoma of the head and neck (Hsueh et al., 2022). Microsatellite instability (MSI) refers to the alteration in the length of microsatellite sequences, which are DNA sequences consisting of short repetitive motifs, during cellular replication. MSI is typically caused by defects in the DNA MMR system, including mutations in MMR genes, epigenetic changes, or other mechanisms. Therefore, the detection of MSI has become an important indicator for assessing tumor risk, diagnosis, and treatment strategy selection. In patients with GC, oral microbiota of oral origin is associated with immune gene expression and tumor mutation burden (Byrd et al., 2023). There is a lack of foundational experimental evidence regarding whether microbiota in GC affects other DNA repair deficiencies. Investigating the disruption of microbiota on DNA repair may hold significant implications for understanding the molecular mechanisms underlying the onset and progression of GC. Microbiota increase host cell genome damage and inhibit genome repair as shown in **Figure 2**.

### 3.3 Microbiota dysbiosis and aberrant signaling pathways in GC

**Figure 3:**
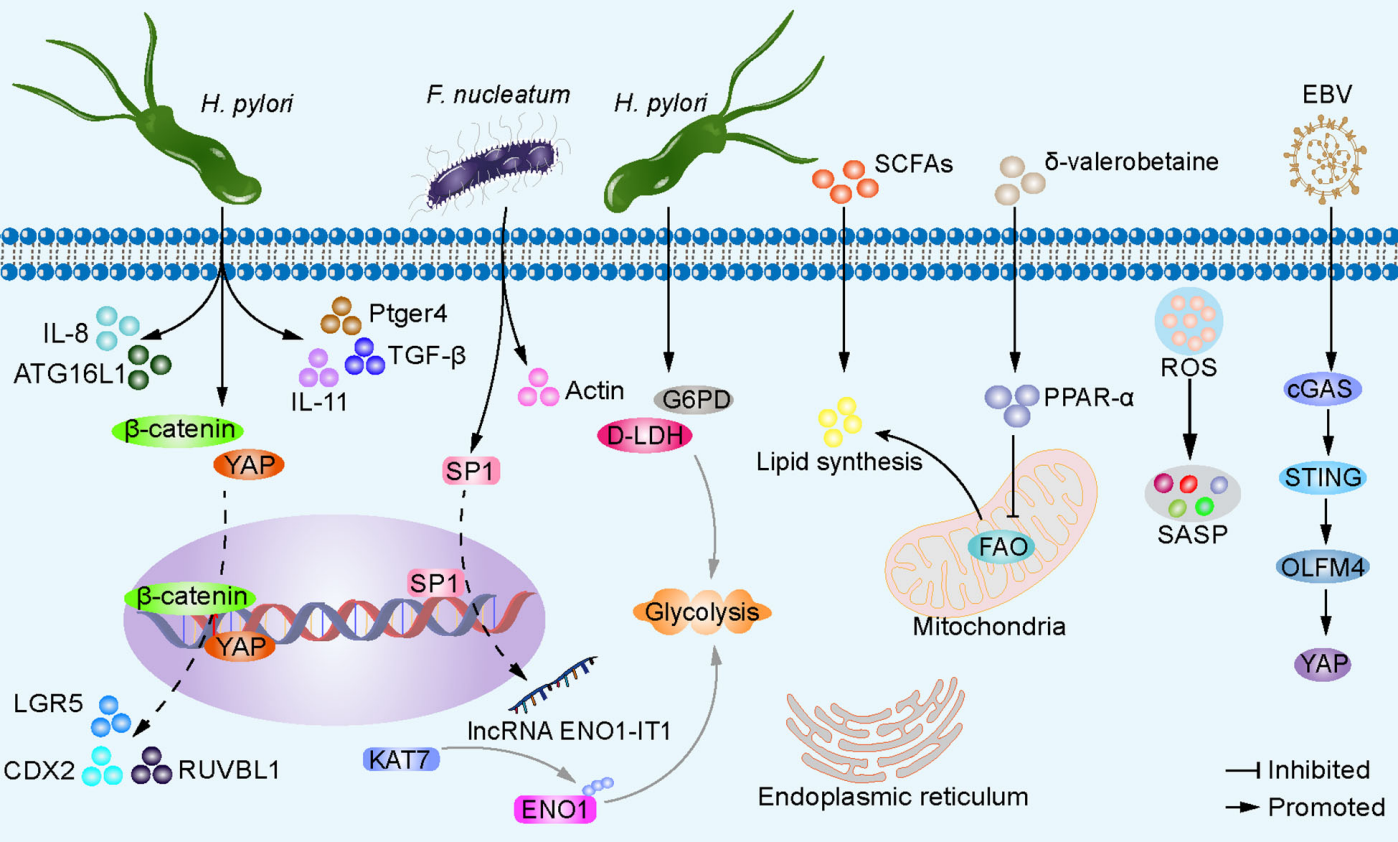
Microbiota dysbiosis alters gastric epithelial cell signaling pathways. *H. pylori* promotes nuclear accumulation and transcriptional activity of YAP and β-catenin in gastric epithelial cells, leading to activation of target genes CDX2, LGR5, and RUVBL1, facilitating cell proliferation and expansion, ultimately resulting in GC development. *H. pylori* also induces the expression of IL-11 and cancer-related genes Ptger4 and TGF-β. *H. pylori* enhances autophagy gene ATG16L1, increasing IL-8 production, driving carcinogenesis. *H. pylori* induces the expression of G6PD and D-LDH in host cells, facilitating glycolysis, and energy production. *F. nucleatum* upregulates transcription factor SP1, activates lncRNA ENO1-IT1 transcription, guides KAT7 histone acetyltransferase to modify target gene ENO1, increasing host cell glycolysis. δ-valerobetaine produced by various bacteria inhibits mitochondrial FAO and increases lipid accumulation via transcription factor PPAR-α. SCFAs serve as substrates for lipid synthesis. Additionally, the microbiota can induce sustained inflammatory responses, generate ROS, causing DNA fragmentation, membrane disintegration, and protein misfolding through modification of key substrates such as nucleic acids, lipids, and proproteins, leading to cellular senescence, secretion of SASPs, and accelerated tumor growth. EBV infection can activate the cGAS-STING pathway and upregulate the expression of OLFM4, thereby leading to the activation of YAP in recipient cells.

The interaction between dysbiosis and cancer cell aberrations involves multiple signaling pathways that can influence tumor initiation, progression, and therapeutic response (**Figure 3**). Infection with *H. pylori* drives the nuclear accumulation and transcriptional activity of yes-associated protein 1 (YAP) and β-catenin in gastric epithelial cells and transgenic insulin-gastrin mice. This interaction between YAP and β-catenin promotes their nuclear activation. Consequently, the activation of target genes such as CDX2, LGR5, and RUVBL1 is initiated, fostering cell proliferation, and contribute to the pathogenesis of GC (Li et al., 2023). *H. pylori* infection not only triggers the expression of IL-11 but also upregulates cancer-related genes such as PTGER4 and TGF-β in insulin-gastrin mice. These molecular changes further expedite the progression of gastric cancer (Lertpiriyapong et al., 2014). *F. nucleatum* induces the activation of actin and genes regulating cell motility, promoting the invasiveness of GC cells (Hsieh et al., 2021). In addition, the microbiota can induce sustained inflammatory responses, generating ROS and causing DNA fragmentation, membrane breakdown, and protein misfolding through modifications of key substrates such as nucleic acids, lipids, and proteins (Chen et al., 2022c). These processes may lead to cellular senescence (Pérez-Mancera et al., 2014). Senescent cells stand apart from quiescent and apoptotic cells by maintaining high cellular viability and efficient metabolic function (Campisi and D’adda Di Fagagna, 2007). Senescent cells collectively produce a range of cytokines, chemokines, growth factors, proteases, and other secretory signaling factors, forming what is known as the senescence-associated secretory phenotype (SASP) (Coppé et al., 2010). Senescent cells have a dual role through autocrine or paracrine signaling: they play a physiological role in tissue development, prevent proliferation of damaged cells, aid in tissue repair, and contribute to tumor suppression. However, they also promote the onset of age-related diseases, including cancer (Chen et al., 2022b). Mounting evidence indicates that dysregulated SASP sustains an inflammatory environment, promoting cancer cell proliferation, migration, invasion, and EMT, thereby accelerating the growth of xenograft tumors (Chen et al., 2022b). Research on the mechanisms of microbial dysbiosis in GC cells remains unclear, presenting a highly promising avenue for investigation. In addition, certain viruses can also trigger the abnormal activation of signaling pathways. For example, Epstein-Barr virus (EBV) infection can activate the cGAS-STING pathway and upregulate the expression of olfactomedin 4 (OLFM4), which binds to the extracellular cadherin domain of FAT1, thereby disrupting its intracellular interaction with MST1 and subsequently activating YAP in recipient cells (Wen et al., 2024). Naturally occurring or genetically engineered viruses, such as the CF33 oncolytic virus, are capable of delivering functional proteins (e.g., hNIS-antiPDL1) and exhibit significant antitumor activity in peritoneal metastasis gastric cancer models following intraperitoneal injection (Yang et al., 2023a). The expression and/or integration of human papillomavirus oncogenes in gastric cancer may play a potential etiological role, but the underlying mechanisms remain to be further explored (Xu et al., 2023a).

The imbalance of the microbial community may lead to abnormal accumulation or deficiency of metabolites, thereby affecting host metabolic health. These metabolites can influence host cell function and metabolic status through different signaling pathways. For example, in atrophic gastritis induced by *H. pylori*, there is an elevated expression of glucose-6-phosphate 1-dehydrogenase (G6PD) and D-lactate dehydrogenase (D-LDH) (Parsons et al., 2017). This contributes to inducing anaerobic metabolic shift, thereby generating energy (Chen et al., 2023c). Some bacteria enriched in GC tissues, although not yet reported in GC, have been shown to alter the glycolipid metabolism of other tumor cells. For example, *F. nucleatum* activates the transcription of long non-coding RNA ENO1-IT1 by enhancing the binding efficiency of transcription factor SP1 to the promoter region of ENO1-IT1. The increased expression of ENO1-IT1 acts as a guiding module for KAT7 histone acetyltransferase, directing its histone modification pattern on target genes, including ENO1, a key glycolytic enzyme, thereby altering glycolysis in colorectal cancer cells (Hong et al., 2021). The microbiota provides lipid synthesis precursors or stimulates host cell lipid synthesis through its own metabolic products. For example, short-chain fatty acids (SCFAs) can serve as substrates for energy production, lipid synthesis, gluconeogenesis, and cholesterol synthesis (Bergman, 1990). δ-Valerobetaine, generated by diverse bacterial strains, activates the transcription factor PPAR-α, thereby driving transcriptional regulation of lipid processing and mitochondrial energy metabolism in the liver of mice. As a result, there is a reduction in mitochondrial fatty acid oxidation (FAO) and an increase in lipid accumulation (Liu et al., 2021). The biologically active components derived from the small bowel microbiota *Clostridium bifermentans* selectively induce the expression of diacylglycerol O-acyltransferase 2 (DGAT2), which participates in triacylglycerol synthesis. The exact mechanism behind this induction remains to be explored (Liu et al., 2021). *H. pylori* can decrease endoplasmic reticulum stress levels in gastric epithelial cells while enhancing the autophagy gene ATG16L1 (rs2241880, G-allele) expression, thereby promoting increased IL-8 production and driving the carcinogenesis process. This may be associated with the role of IL-8 recruitment of granulocytes in the development of intestinal metaplasia and GC (Fu et al., 2016; Mommersteeg et al., 2022). Investigating dysregulated microbiota and abnormal signaling pathways in GC enhances our comprehension of tumorigenesis mechanisms. This exploration sheds light on the microbiota’s involvement in GC development, offering novel insights and strategies for GC prevention, diagnosis, and treatment.

### 3.4 Microbiota and GC hypoxia

Hypoxia is considered a hallmark of cancer, with most solid tumors, including GC, exhibiting oxygen deficiency (Ye et al., 2019). The intratumoural microbiota is intricately associated with hypoxia in TME. This linkage is evident through several avenues: Microbial colonization within tumor tissues elicits inflammatory responses, leading to endothelial cell injury, dysfunctional endothelial cell activity, and compromised vascular function, ultimately culminating in hypoxia due to impaired blood perfusion. Certain microbes, such as *H. pylori* and *Streptococcus* spp., which are facultative anaerobes or aerobes, further contribute to hypoxia by consuming oxygen (Cullin et al., 2021; Chen et al., 2022c). Mechanistically, bacteria can utilize high-affinity terminal oxidases to scavenge O_2_ at low concentrations, even at nanomolar levels, exacerbating the degree of hypoxia in tumor tissues (Morris and Schmidt, 2013; Kelly et al., 2015). Furthermore, microbial derivatives such as SCFAs increase oxygen consumption by pathways including β-oxidation of butyrate and oxidative phosphorylation-dependent epithelial O_2_ consumption (Hamer et al., 2008; Kelly et al., 2015; Zheng et al., 2017). Additionally, microbial communities recruit innate and adaptive immune cell infiltrates, most notably neutrophils and eosinophils, which consume local oxygen via the NADPH oxidase-2 (NOX-2) during oxidative bursts (Campbell et al., 2014; Masterson et al., 2019) Therefore, the microbial community in TME is one of the factors contributing to the formation and maintenance of chronic hypoxia, driving alterations in tumor cell signaling pathways, primarily associated with increased expression of the hypoxia-inducible factor (HIF). This is associated with tumor size, lymph node involvement, vascular invasion, and pathological staging (Zhang et al., 2010). In mice infected with *H. pylori*, levels of HIF-1α significantly increase, enhancing the toxicity of CagA, promoting IL-8 secretion, and exacerbating host pro-inflammatory responses (Noto et al., 2023). Conversely, the massive production of ROS generated by inflammatory responses not only stimulates the expression of HIF-1α but also contributes to its stabilization under hypoxic conditions (Leung and Chan, 2009). TLRs are a highly conserved class of pattern recognition receptors that detect pathogen-associated molecular patterns and play a crucial role in the immune system, protecting the body from infections by initiating immune responses (Akira and Takeda, 2004). LPS activates the tumor cell TLR4 signaling pathway and NF-κB, thereby upregulating HIF-1α, promoting the progression of pancreatic adenocarcinoma (Zhang et al., 2010). Activation of HIF reprograms metabolism, protein synthesis, and cell cycle processes (Chen et al., 2023c).

### 3.5 Microbiota and tumor immune microenvironment

**Figure 4:**
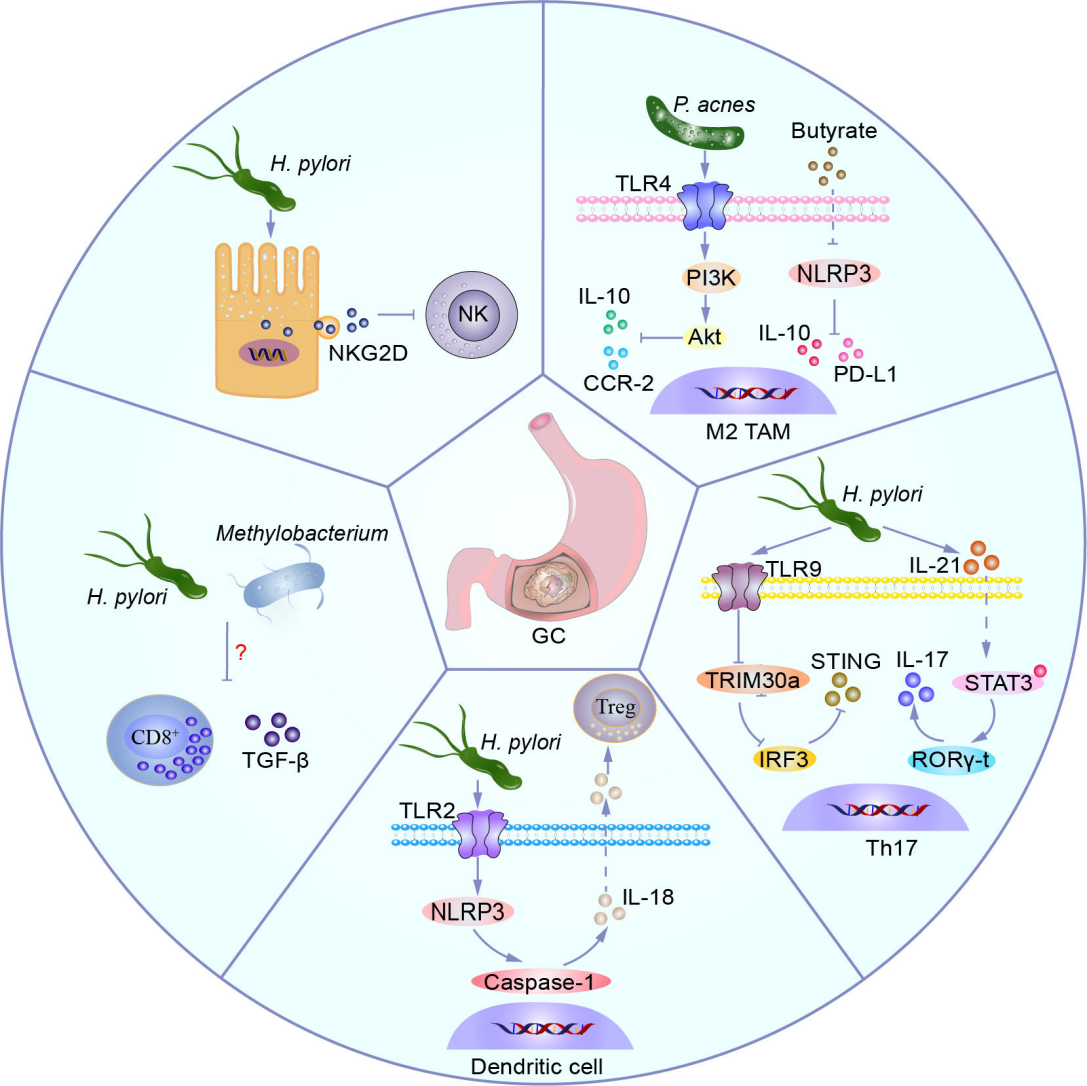
Microbiota shapes the suppressive immune microenvironment. *H. pylori* induces expression of the NKG2D ligand in gastric epithelial cells, which is released from the cell surface via protein hydrolysis or extracellular vesicles, leading to decreased expression of the NKG2D receptor on NK cells and cytotoxic granule degranulation, thereby facilitating immune evasion by tumor cells. *P. acnes* activates the TLR4/PI3K/Akt signaling pathway, inducing M2 TAM polarization, promoting secretion of immunosuppressive factors IL-10 and CCR-2. Butyrate, a derivative of probiotics, negatively regulates the NLRP3-mediated inflammatory signaling pathway, inhibits related macrophage activation, and decreases levels of PD-L1 and IL-10 expression, thereby suppressing tumor growth in mice. *H. pylori* activates TLR9, promotes expression of negative feedback regulator TRIM30a, downregulates activation of transcription factor IRF3, inhibits the STING signaling pathway, and promotes Th17 inflammatory responses and anti-tumor responses *in vivo*. *H. pylori* drives activation of pro-inflammatory T cells, secretes IL-21, induces phosphorylation of STAT3, and induces expression of RORγ-t, promoting Th17 differentiation and IL-17 secretion. *H. pylori* activates dendritic cells via the TLR2/NLRP3/caspase-1/IL-18 axis to induce Tregs, shaping the immune suppressive microenvironment. *H. pylori* and *Methylobacterium* can reduce expression of TGF-β and CD8^+^ T cell infiltration in a GC mouse model, but their mechanisms remain to be elucidated.

A wealth of evidence suggests that dysbiosis of the gastric microbiota and immune system dysfunction, particularly immune evasion, are critical for the onset and progression of GC. Changes in the recruitment and function of innate and adaptive immune cells predominantly drive the progression and prognosis of GC. *H. pylori* induces the expression of natural killer group 2, member D (NKG2D) ligands on gastric epithelial cells through vacA, which are released from the cell surface via protein hydrolysis or extracellular vesicles (EVs). This leads to downregulation of the NKG2D receptor expression on NK cells and cytotoxic granule release, thereby contributing to immune evasion by tumor cells (Anthofer et al., 2024). *P. acnes* significantly increases in GC tissues infected with *H. pylori*, activating the TLR4/PI3K/Akt signaling pathway, inducing polarization of M2-type tumor-associated macrophages (TAMs), and promoting the secretion of immunosuppressive factors IL-10 and CCR-2 (Li et al., 2021c). M2 TAMs maintain an inflammatory environment in TME, creating an immunosuppressive microenvironment that promotes tumor cell proliferation and survival, fosters cancer stem cells, supports metastasis, and contributes to the progression and metastasis of GC (Mantovani et al., 2017; Long et al., 2019; Piao et al., 2022). Butyrate derivatives from probiotics negatively regulate the NLRP3-mediated inflammatory signaling pathway, inhibit the activation of associated macrophages, and reduce their expression levels of PD-L1 and IL-10, thereby suppressing tumor growth in mice (Yao et al., 2022; Lee et al., 2024). *H. pylori* and *Methylobacterium* can decrease the TGF-β expression and infiltration of CD8^+^ T cells in GC mouse models, but their mechanisms remain to be elucidated (Oster et al., 2022; Peng et al., 2022). Some less abundant bacterial genera in GC tissues, such as *Selenomonas* and *Brevundimonas*, are positively correlated with regulatory T cells (Tregs) (Ling et al., 2019; Yang et al., 2022a). Mechanistically, *H. pylori* activates the TLR2/NLRP3/caspase-1/IL-18 axis in dendritic cell to induce Tregs, shaping an immunosuppressive microenvironment (Koch and Müller, 2015). *H. pylori* drives the activation of pro-inflammatory T cells, secretes IL-21, induces STAT3 phosphorylation, and promotes RORγ-t expression, facilitating the differentiation of T helper 17 (Th17) cells and the secretion of IL-17 (Carbo et al., 2014). Additionally, *H. pylori* activates TLR9, promotes the expression of the negative regulatory factor TRIM family protein TRIM30a, thereby downregulating the activation of transcription factor interferon regulatory factor 3 (IRF3) and inhibiting the stimulator of interferon genes (STING) signaling pathway. These mechanisms contribute to the induction of Th17 cell inflammatory response and tumor-promoting effects *in vivo* (Dooyema et al., 2022). *Candida* is positively correlated with pro-inflammatory immune factors IL1A, IL1B, IL6, IL8, CXCL1, CXCL2, and IL17C, which are associated with neutrophil and Th17 cell infiltration (Dohlman et al., 2022; Li et al., 2022). These possible mechanisms are summarised in **Figure 4**.

Furthermore, dysbiosis of the microbiota may affect other stromal cells in the tumor microenvironment, such as endothelial cells. Dysbiosis of the microbiota can disrupt the balance between pro-angiogenic and anti-angiogenic factors, crucial for angiogenesis. This imbalance may accelerate tumor angiogenesis, leading to rapid but abnormal blood vessel formation (Carmeliet and Jain, 2000; Jain, 2005; 2014). *In vitro*, low concentrations of probiotic metabolite butyrate promote angiogenesis via G-protein-coupled receptor 43 (GPR43, also known as FFAR2) (Castro et al., 2021). LPS stimulation of NOD-like receptor (NLR) and TLR increases microvascular formation, inducing human intestinal microvascular endothelial cell migration and proliferation (Peng et al., 2004). Nevertheless, dysbiosis of the microbiota plays a vital role in various critical aspects of GC development. Particularly, the enrichment of intratumoral probiotics and their metabolites in GC warrants further exploration. The σC protein from avian reovirus or UV-inactivated avian reovirus can bind to TLR3 on the surface of CD8^+^ tumor-infiltrating lymphocytes, activating the TLR3/NF-κB/IFN-γ/TRAIL signaling pathway in immune cells. This induces the production of TRAIL, thereby initiating immunogenic apoptosis targeting cancer cells (Wu et al., 2024b). Based on these mechanisms, microbial dysbiosis affects GC chemotherapy and immunotherapy (Li et al., 2021b; Kim et al., 2023; Magahis et al., 2023). Exploring the microbial mechanisms of carcinogenesis helps deepen our understanding of the interplay between microbiota and GC development, providing new research perspectives and strategies for both preventing and treating GC.

Although this study systematically explores several key molecular mechanisms (such as CagA, short-chain fatty acids, DNA repair pathways, and the IL-6/JAK/STAT3 signaling axis), it lacks in-depth analysis linking these mechanisms to clinical practice. Their potential value in diagnostic biomarker development, prognostic assessment, therapeutic target identification, and resistance mechanisms has not been fully demonstrated. Future research could further investigate the clinical applicability of these mechanisms, such as their roles in biomarker screening, patient stratification, and prediction of treatment response, in order to enhance the translational relevance and clinical impact of the study.

## 4 Microbiota-related therapeutic application

**Figure 5:**
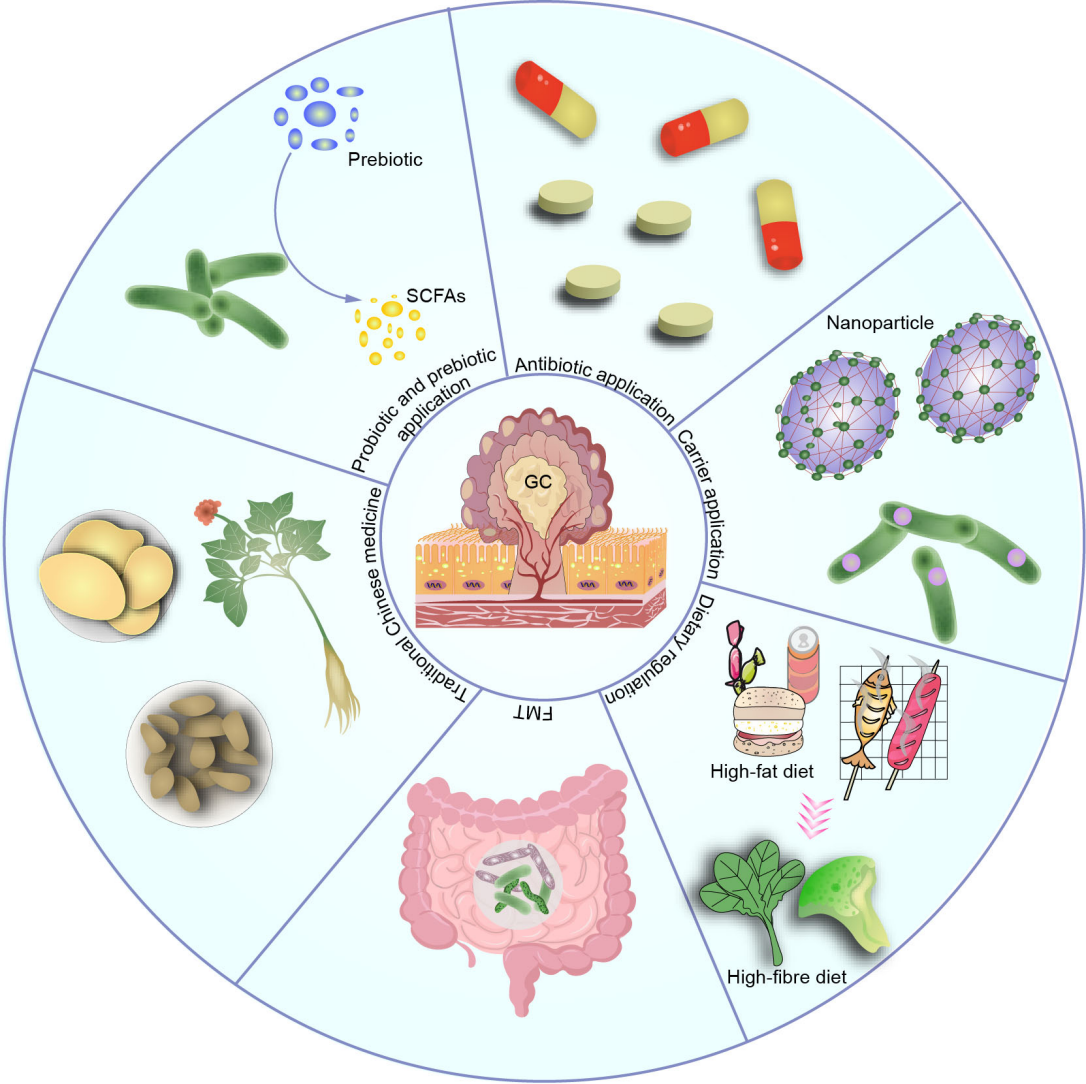
Therapeutic applications based on the microbiota, such as probiotic, prebiotic, antibiotic use, carrier application, dietary modulation, and traditional Chinese medicine, have shown promising efficacy. However, most of these applications are still in the preclinical stage, and their clinical efficacy and potential complications remain to be determined.

The application of microbiota-related therapy in GC is gradually becoming a focus of research. These therapeutic approaches include probiotic therapy, prebiotic therapy, antimicrobial therapy, carrier application, dietary adjustments, fecal microbiota transplantation (FMT), and traditional Chinese medicine treatment, aiming to regulate gut microbiota balance, improve intestinal health in GC patients, and enhance immune system function (**Figure 5**). Although these therapeutic methods are still in the research and exploration stages, they offer new insights and hope for the treatment of GC.

### 4.1 Probiotic application

The application of probiotics in GC treatment is an area of great interest. Research indicates that probiotics and their derivatives can impact the onset and progression of GC by altering the gut microbiota, influencing the host’s immune status, and regulating inflammation levels (Cao et al., 2022). On one hand, probiotics can improve the structure of the microbiota, particularly in the post-GC surgery gut microbiota, enhancing host immunity (Zheng et al., 2019, 2021a; He et al., 2022). This helps to ameliorate intestinal dysbiosis caused by mechanical bowel preparation, thereby reducing the incidence of postoperative delirium (Yang et al., 2022c). Furthermore, probiotics and their derivatives have been found to alleviate intestinal damage induced by chemotherapy drugs like oxaliplatin in both mice and human patients. They also enhance the response to anti-programmed cell death protein 1 (PD-1)/programmed death-ligand 1 (PD-L1) immunotherapy (Yuan et al., 2022; Han et al., 2023). On the other hand, probiotic derivatives such as butyrate salts enhance the cytotoxic function of CD8^+^ T cells or CAR-Claudin 18.2 CD8^+^ T cells against GC cells via GPR109A and homeodomain-only protein X (HOPX) (Yu et al., 2024). A combination of probiotics (*Lactobacillus acidophilus NCFM* and *Lactobacillus plantarum Lp-115*) effectively recruits more lymphocytes, plasma cells, and neutrophils (Shen et al., 2023; Ye et al., 2024). Furthermore, probiotics such as *Lactobacillus* significantly reduce inflammatory cytokines, preventing host macrophages from producing pro-inflammatory cytokines IL-1β, IL-6, IL-8, TNF-α, and IFN-γ (Gebremariam et al., 2019; Zhao et al., 2022; Wu et al., 2023). *Lactobacillus rhamnosus GG* induces FPR1, a tumor suppressor, to maintain inflammation resolution with anti-angiogenic potential (Liotti et al., 2022). However, the heightened presence of *lactobacillus* during cancer progression challenges the notion of their predominantly protective role in GC. *Lactobacillus* contributes to carcinogenesis by promoting factors such as ROS, N-nitroso compounds, lactate production, as well as inducing EMT and immune tolerance (Yang et al., 2021; Nabavi-Rad et al., 2022). Therefore, due to the unclear roles and functionalities of some probiotics enriched in GC tissues, the application of probiotics in GC patients needs to be approached with caution.

### 4.2 Prebiotic application

Prebiotics, indigestible substances metabolized by probiotic bacteria into SCFAs like acetate, propionate, and butyrate, play a vital role in promoting human health. They enhance resistance to pathogenic colonization, maintain mucosal barrier integrity, regulate intestinal pH, and boost anti-tumor immunity, thereby enhancing anti-cancer activity (Verspreet et al., 2016). The combination of sodium butyrate and dexamethasone significantly downregulates the oncogene TNS4 in GC cells, exhibiting a notable anti-proliferative effect (Eladwy et al., 2024). Raffinose is a polysaccharide composed of one molecule of glucose, one molecule of galactose, and one molecule of fructose. It is particularly abundant in foods such as beans, onions, beets, and carrots. Human gastrointestinal tract cannot directly digest and absorb raffinose, but it is fermented by the microbes in the intestine, producing SCFAs, which can lower the risk of GC (Turati et al., 2023). Ellagic acid is a bioactive phytochemical known for its high antioxidant and anticancer effects. However, its absorption rate in the intestine is low, and it is easily excreted. When encapsulated with low methoxylated and high methoxylated pectin films at a 1:4 molar ratio, ellagic acid lysine salt not only increases the water solubility of ellagic acid but also preserves its biological activity. After fermentation by gut microbiota, it produces SCFAs, demonstrating good prebiotic activity (Ortenzi et al., 2021). Some prebiotics, such as mushroom polysaccharides, can stimulate the growth of beneficial bacteria in the colon (Nowak et al., 2018). Combination formulations containing both probiotics and prebiotics are promising for promoting intestinal health, enhancing immune function, and improving nutrient utilization. More research is necessary to fully comprehend the role and functionality of prebiotics in mitigating the risk of digestive tract tumors (Enache et al., 2022). In addition, some postbiotics, such as Urolithin A, not only exert their anti-tumor effects by activating autophagy and further activating the downstream Hippo pathway, inhibiting the Warburg effect, and promoting cell apoptosis, but also by modulating the composition of the gut microbiota, resulting in an increase in probiotics and a decrease in pathogenic bacteria (Qiao et al., 2024).

### 4.3 Antibiotic application

Antibiotics have the potential to impact tumors through altering the microbiota, modulating immune responses, and affecting their own drug metabolism. Co-administration of antibiotics with probiotics can reduce the changes and imbalance in the intestinal microbiota induced by antibiotics and improve the success rate of eradicating *H. pylori* (Oh et al., 2016) However, long-term antibiotic use may increase the risk of cancer development (Boursi et al., 2015; Hao et al., 2022; Chen et al., 2023b), or lead to complications such as anemia, gastrointestinal bleeding, and mortality (Quinn et al., 2020). For example, in multiple cohorts of patients with advanced GC undergoing PD-1 inhibitor therapy, the use of antibiotics has consistently been associated with poorer PFS and OS (Kim et al., 2023). Additionally, existing antibiotic-based traditional approaches lack targeted effects, resulting not only in failure in approximately 20% of patients but also in severe bacterial resistance and disruption of gut microbiota. This may be associated with upregulation of multidrug resistance proteins, methicillin-resistant regulator proteins, vancomycin-resistant sensor histidine kinases, chloramphenicol resistance proteins, and tetracycline resistance proteins (Guo et al., 2020). Therefore, the development of alternative or antibacterial agents is crucial for treating GC. Nanostructured lipid carriers (NLC), even when not loaded with any drugs, show bactericidal effects against *H. pylori* at low concentrations. Mechanistically, NLC can rapidly bind to and penetrate the membrane of *H. pylori*, causing destabilization and disruption. This leads to the leakage of cytoplasmic contents and ultimately results in bacterial death (Seabra et al., 2018; Chitas et al., 2022). A pH-responsive metal-organic framework hydrogen-generation nanoparticle (Pd(H)@ZIF-8) encapsulated in an ascorbate palmitate (AP) hydrogel can target and adhere to inflammatory sites through electrostatic interactions. Subsequently, it undergoes hydrolysis by matrix metalloproteinases. The released Pd(H)@ZIF-8 nanoparticles are further decomposed by gastric acid, producing zinc ions (Zn^2+^) and hydrogen gas. This process effectively kills *H. pylori*, alleviates inflammation, and helps restore damaged gastric mucosa. Additionally, this approach helps to avoid dysbiosis of the intestinal microbiota (Zhang et al., 2022). It’s interesting that cancer risk, including GC, is reduced in diabetic patients treated with metformin. Metformin exhibits direct antibacterial activity against *H. pylori*, but its widespread applicability and mechanism require further elucidation (Jauvain et al., 2021). Engineering common dairy probiotics like *Lactobacillus* into complexes that secrete *H. pylori*-binding guide peptide (MM1) and broad-spectrum antimicrobial peptides can offer high selectivity against *H. pylori* while avoiding the development of pathogen resistance (Choudhury et al., 2023).

### 4.4 Carrier application

Some microbiota can metabolize chemotherapy drugs, greatly reducing their bioavailability. Therefore, carriers act as a medium, delivering drugs to specific areas through their own specific biological functions or by carrying substances with biological functions. Common carriers include NPs and biological carriers. Encapsulating 5-fluorouracil (5-FU) in chitosan NPs (CS NPs) and incorporating them into retrograde starch and pectin (RS/P) microparticles can prevent premature degradation or release of the NPs as they pass through the stomach and upper digestive tract, ensuring that 5-FU reaches the colon (Dos Santos et al., 2021). Rhamnogalacturonan-I is a type of natural pectic polysaccharide. When passing through gastric and intestinal fluids, capsules exhibit minimal *in vitro* release, degrading only through the action of colonic microbiota. Leveraging this property, the substance can serve as an excellent carrier for drug delivery (Svagan et al., 2016). Probiotics coated with silk fibroin NPs or mineralized coatings can prevent damage in the stomach, enhance survival rates, reach the intestine, regulate the gut microbiota, and synergistically enhance therapeutic effects in a mouse model of intestinal mucosal inflammation (Hou et al., 2021; Geng et al., 2023). Conversely, probiotics can also serve as oral drug carriers, transporting medications (such as metal NPs) to the intestines. This not only enhances the gut microbiota but can also be utilized for magnetic hyperthermia and photothermal therapy (Garcés et al., 2022). Furthermore, some rare elements such as selenium have beneficial effects on intestinal inflammation after trace intake. Constructed Se@Albumin complex NPs significantly ameliorate chemotherapy-induced complications of intestinal mucositis in a mouse model by reducing intestinal oxidative stress levels, lowering intestinal permeability, and alleviating gastric motility disorders (Deng et al., 2021). A pH-responsive ROS nanogenerator (Fe-HMME@DHA@MPN) consists of an acid-responsive metal phenolic network (MPN) shell and a mesoporous metal-organic nanostructure core [Fe-HMME (hematoporphyrin monomethyl ether, a sonosensitizer)]. Encapsulating dihydroartemisinin (DHA), these NPs generate more ROS singlet oxygen under ultrasound than the sonosensitizer HMME alone. The sonochemical process is driven by the Fenton/Fenton-like reaction between the degradation product Fe (II) in gastric acid and hydrogen peroxide (H_2_O_2_) in the infected microenvironment, producing oxygen. Encapsulated DHA acts as a hydrogen peroxide source, enhancing the peroxidase-like activity of Fe-HMME@DHA@MPN, thereby generating ROS hydroxyl radicals to kill multidrug-resistant Helicobacter pylori and eradicate biofilms, with minimal effects on the normal gut microbiota (Yu et al., 2023).

### 4.5 Dietary regulation

Dietary habits can influence the occurrence and development of GC by modulating the microbiota. Diet represents an economical, non-invasive, natural, and sustainable therapeutic approach. On one hand, a Western diet, characterized by high fat, high sugar, and low fiber intake, can disrupt the balance of gut microbiota, a condition known as dysbiosis. This dysbiosis contributes to an increased risk of GC (Rinninella et al., 2020). The high-fat diet leads to severe dysbiosis in the stomach. Changes in the microbiota are accompanied by an increase in gastric leptin, leading to the development of intestinal metaplasia (Arita and Inagaki-Ohara, 2019; Arita et al., 2019). Typically, high-temperature (150-300°C) cooking and nitrite-curing of meats result in the formation of toxic compounds like heterocyclic amines. These compounds have a high mutagenic potential and are implicated in the development of colon cancer and GC. *Lactobacillus casei DN 114001* reduces the genotoxicity of heterocyclic amines, suggesting that bacteria may metabolize or adsorb heterocyclic amines (Nowak and Libudzisz, 2009; Van Hecke et al., 2015). Recent studies suggest that a diet rich in capsaicin, the primary pungent compound in chili peppers, might promote gastric cancer metastasis. This effect could occur through the regulation of transient receptor potential vanilloid 1 (TRPV1) expression and alterations in the gut microbiota composition. This suggests the importance of controlling chili consumption for GC patients (Deng et al., 2023). On the other hand, a high-fiber diet, foods rich in probiotics and prebiotics, and other similar dietary choices can foster the growth of beneficial bacteria and help maintain the balance of intestinal microbiota, thereby reducing the risk of developing GC (Rinninella et al., 2020). Some foods contribute to the growth of probiotics. For example, spinach rich in cobalamin is positively correlated with genera of *Bacteroides*, propionates, and butyrates (Zheng et al., 2021b). Vegetable and seafood patterns may interact with dysbiosis to mitigate the risk of male GC, while dairy patterns may interact with dysbiosis to reduce the risk of GC in females (Gunathilake et al., 2021). Based on exogenous metabolites, adenosylcobalamin, soybean, common wheat, dates, and barley are considered potential candidates for the treatment of atrophic gastritis without *H. pylori* infection, while gallate from gallnuts is considered a candidate for the treatment of atrophic gastritis with *H. pylori* infection (Gao et al., 2023). Dairy products containing baicalin and baicalein can inhibit the expression of the vacA gene in *H. pylori*, interfere with its adhesion and invasion capabilities to human GC cells, and reduce the levels of *H. pylori*-specific serum IgM and IgA as well as IL-8 expression (Chen et al., 2018). Taken together, adjusting the dietary habits of GC patients to modulate the microbiota offers multiple advantages, including safety, comprehensiveness, naturalness, sustainability, and comprehensiveness. This approach represents an effective means of preventing and supporting the treatment of GC.

### 4.6 FMT

FMT not only alters the composition of bacteria but also establishes a cross-domain balance between intestinal fungi, viruses, and bacteria to promote the restoration of microbial homeostasis. Prior to first-line chemotherapy, FMT from healthy obese donors may improve the chemotherapy response (to capecitabine and oxaliplatin) and survival rates of patients with metastatic esophagogastric cancer (De Clercq et al., 2021). After radical gastrectomy, patients undergoing FMT exhibit immunomodulatory effects by adjusting the intestinal microbiota structure, characterized by an increase in the relative abundance of certain bacteria producing SCFAs. Mechanistically, butyrate downregulates the NLRP3-mediated inflammatory signaling pathway, inhibits macrophage activation, and suppresses the secretion of pro-inflammatory mediators such as cysteine aspartate-specific protease-1 and IL-1β, thereby reducing intestinal inflammation levels and promoting nutrient absorption (Yao et al., 2022). FMT is an effective treatment for recurrent *Clostridium difficile* infection, with its effectiveness in preventing recurrence reaching approximately 90% (Konturek et al., 2015). However, FMT may lead to complications that should not be overlooked, including the possibility of pathogen transmission to the recipient. Therefore, FMT is not a one-size-fits-all approach, and research is needed to determine the microbial composition that has specific effects on patients with different diseases. *Akkermansia muciniphila* can enhance the anticancer effect of oxaliplatin by producing pentadecanoic acid, which inhibits the activity of the glycolysis regulator far upstream element binding protein 1, thereby blocking aerobic glycolysis in cancer cells (Xu et al., 2024). This suggests that FMT can alter the gut microbiota structure, thereby enhancing the potential efficacy of chemotherapeutic agents, such as oxaliplatin, in GC.

### 4.7 Traditional Chinese medicine

Traditional Chinese medicine can influence the progression of cancer by regulating the gut microbiota. It alters the composition and structure of the gut microbiota and modifies the levels of endogenous metabolites. These changes enhance intestinal barrier function, bolster the immune system, and improve overall body metabolism, contributing to the significant anti-tumor properties of Chinese herbal medicine (Wei et al., 2024). Jianpi Yangzheng helps regulate the structure of the gut microbiota and reduces the proportion of myeloid-derived suppressor cells, along with their production of inflammatory factors (Zhu et al., 2024). Gexia-Zhuyu Tang inhibits GC growth, reduces the expression levels of proteins associated with metastasis and invasion, including CD147, vascular endothelial growth factor (VEGF), and matrix metalloproteinase-9 (MMP-9). Additionally, modified Gexia-Zhuyu Tang significantly enhances caspase-1-dependent pyroptosis. This is supported by a dose-dependent rise in TNF-α, IL-1β, IL-18, and lactate dehydrogenase (LDH) levels, accompanied by increased protein expression of NLRP3, apoptosis-associated speck-like protein (ASC), and caspase-1 (Zhao and Yu, 2024). Cordycepin has antibacterial and anti-inflammatory effects on mice infected with *H. pylori*. Compared to the control group treated with the carrier alone, cordycepin treatment results in approximately 50% reduction in the production of inflammatory cytokines, including IL-6 and IL-1β, and about 60% reduction in the infiltration of immune cells such as Th17 cells (Kong et al., 2022). Therefore, oral traditional Chinese medicine exhibits multiple effects such as anti-tumor properties, immune modulation, alleviation of side effects, and improvement of overall health. Although the efficacy of traditional Chinese medicine in treating GC requires further scientific validation and clinical research, its role as an adjunctive therapy holds promise in enhancing patient quality of life and mitigating treatment side effects. The mechanisms, outcomes, and limitations of microbiota-related therapeutic applications are summarized in **Table 2**.

**Table 2:** Mechanisms, outcomes, and limitations of Microbiota-related therapeutic applications.

Microbiota-related therapeutic application	Mechanisms	Outcomes	Limitations
Probiotic application	Regulate gut microbiota structure and restore balance after GC surgery. Enhance host immunity and CD8^+^ T/CAR-T cell cytotoxicity. Reduce inflammation by lowering IL-1β, IL-6, TNF-α, and mitigating chemo-induced gut damage. Improve response to PD-1/PD-L1 immunotherapy. Recruit immune cells via specific probiotic combinations.	Promote postoperative recovery and reduce delirium by enhancing gut barrier and immunity. Alleviate chemotherapy-induced gut toxicity. Boost PD-1/PD-L1 immunotherapy response. Improve tumor immune microenvironment by enhancing anti-tumor immunity and reducing inflammation.	Some probiotics like *Lactobacillus* may promote GC by inducing ROS, EMT, and immune tolerance. Their roles remain unclear, and effects may be double-edged. Probiotic use in GC should be cautious and strain-specific.
Prebiotic application	Prebiotics boost SCFA production, lower gut pH, suppress harmful bacteria, strengthen the barrier, and enhance anti-tumor immunity. They also promote beneficial bacteria and microbiota stability. Encapsulation improves the stability and efficacy of anti-cancer compounds.	Sodium butyrate with dexamethasone enhances anti-cancer effects in GC. Prebiotics like raffinose and tannic acid boost SCFA production and reduce GC risk. Modified tannic acid shows strong prebiotic activity. Synbiotics offer synergistic benefits for gut and immune health.	Individual gut microbiota differences lead to variable prebiotic effects. Some compounds have low bioavailability before encapsulation. Anti-cancer mechanisms remain under-researched, with limited clinical validation. Prebiotics may also interfere with cancer treatments.
Antibiotic application	Antibiotics combined with probiotics can reduce dysbiosis and improve *H. pylori* eradication. New strategies include NLCs that disrupt bacterial membranes, Pd(H)@ZIF-8 nanoparticles that release Zn²^+^ and hydrogen to kill *H. pylori* and reduce inflammation, and engineered probiotics that selectively target *H. pylori* without promoting resistance.	Antibiotics can effectively eradicate *H. pylori* and combining with probiotics improves success rates. However, long-term use may increase GC risk and reduce immunotherapy efficacy. New approaches like nanocarriers and engineered probiotics offer better targeting and lower resistance.	Long-term use can lead to dysbiosis, an increase in resistant strains, and poor targeting. When combined with immunotherapy (e.g., PD-1 inhibitors), it may reduce efficacy.
Carrier application	Nanoparticles encapsulating chemotherapy drugs can prevent premature release of the drugs in the stomach and upper digestive tract, ensuring they reach the colon to exert their effects. Protecting probiotics through gastric acid increases their colonization rate and improves their therapeutic effect on intestinal mucosal inflammation. Mechanisms such as reducing intestinal permeability help improve chemotherapy-induced intestinal mucosal damage.	The nanodelivery system delays drug release, improves targeting, and enhances therapeutic efficacy. Alleviates chemotherapy-related intestinal toxicity and promotes recovery. Effectively kills resistant bacteria and maintains the stability of the normal gut microbiota.	Primarily animal models, lacking clinical research validation. Complex formulation technology, high production costs, and challenges in large-scale application. The long-term safety, metabolic process, and impact of nanomaterials on the microecosystem are not yet fully understood. Individual differences in gut microbiota may affect drug efficacy and response, requiring personalized strategies for support.
Dietary regulation	Western diets and high-temp cooking disrupt gut microbiota, increasing GC risk. Some compounds (e.g., capsaicin) may promote GC, while foods like spinach and dairy support beneficial bacteria. Veggie/seafood diets may lower GC risk in men; dairy diets may help women.	Western diets and processed meats increase GC risk, while fiber, probiotics, and prebiotics reduce it. Foods like spinach, soybeans, and dates promote beneficial bacteria, lowering GC risk. Dairy products with baicalin and baicalein inhibit *H. pylori* and reduce GC incidence.	Dietary responses vary across populations, influenced by genetics and lifestyle. Most studies are observational, lacking long-term trials to confirm dietary impacts on GC. Due to GC’s multifactorial nature, diet alone may have limited effectiveness, requiring combination with other therapies.
FMT	FMT restores gut microbiota balance in GC patients by promoting SCFA-producing bacteria, which modulate the immune system. Butyrate downregulates the NLRP3 inflammatory pathway, inhibits macrophage activation, and reduces intestinal inflammation, improving nutrient absorption.	FMT improves chemotherapy responses and survival rates in some cancer patients, including those with metastatic esophagogastric cancer. It is highly effective in preventing recurrent *Clostridium difficile* infections (90% success rate) and helps regulate gut microbiota and function.	While FMT shows benefits, it carries risks, such as potential pathogen transmission. It is not suitable for all patients, and personalized treatment based on individual conditions is needed. Further research is required to identify effective microbial compositions for different diseases.
Traditional Chinese medicine	Traditional Chinese medicine impacts gut microbiota composition and metabolism, improving intestinal barrier function, immunity, and metabolism. Herbal formulas like Jianpi Yangzheng and Gexia-Zhuyu Tang inhibit gastric cancer growth by regulating microbiota and immune responses, enhancing pyroptosis. Ingredients like cordycepin reduce inflammation and immune cell infiltration in GC through antibacterial and anti-inflammatory effects.	Traditional Chinese medicine has anti-tumor properties, modulates the immune system, reduces tumor invasion and metastasis, and improves patients’ quality of life. It serves as an adjunct therapy for GC, reducing inflammation, alleviating chemotherapy side effects, and regulating the gut microbiota to improve immune and intestinal function.	The efficacy of traditional Chinese medicine for GC requires more scientific research and clinical trials. Its mechanisms are not fully understood, and more studies are needed to explore its specific actions. Current data is insufficient to support its independent use, and more clinical evidence is necessary.

## 5 Conclusions and perspectives

Although these processes have been extensively studied for decades, the potential impact of the microbiome on cancer development, progression, and treatment response has remained elusive until recently. The microbiota is diverse, abundant, and influenced by factors such as altitude, climate, diet, host immunity, GC heterogeneity, and surgical procedures (Ravegnini et al., 2020; Li et al., 2021a; Jang et al., 2022). This complexity complicates the use of the microbiome in precision therapeutics. Therefore, our primary goal is to identify differentially abundant taxa more accurately. Developing effective metatranscriptomic strategies is crucial for accurately characterizing the microbiome in human tissues with lower microbial biomass, which plays a significant role in microbiome research (Pereira-Marques et al., 2024). Furthermore, we should delve deeper into the specific molecular mechanisms of differential taxa in TME, targeting both tumor and stromal cells. This will help us better understand the roles and functions of microbes in tumor progression, facilitating the development of drugs targeting these key points. Finally, microbial-related therapies await further development. Although some therapeutic applications, such as probiotics, prebiotics, antibiotic applications, carrier applications, dietary regulation, traditional Chinese medicine, and bacteriophages, have shown promising efficacy, most of these are in preclinical stages and come with some significant side effects that cannot be overlooked in GC patients (Federici et al., 2022). Currently, only a small number of clinical trials (such as NCT06250075, NCT05901779, NCT05544396) are underway regarding the clinical investigation of probiotics on the gut microbiota of GC patients, neoadjuvant chemotherapy, and the progression mechanisms of GC. These clinical trials are in either the “enrolling by invitation” or “recruiting” phase, and the therapeutic efficacy and potential complications remain to be determined. In conclusion, research on the microbiota in GC has not only deepened our understanding of this disease but also provided new hope and directions for future treatments.
